# Review of Geopolymer Nanocomposites: Novel Materials for Sustainable Development

**DOI:** 10.3390/ma16093478

**Published:** 2023-04-29

**Authors:** Anna Drabczyk, Sonia Kudłacik-Kramarczyk, Kinga Korniejenko, Beata Figiela, Gabriel Furtos

**Affiliations:** 1 Department of Materials Engineering, Faculty of Materials Engineering and Physics, Cracow University of Technology, 37 Jana Pawła II Av., 31-864 Cracow, Poland; anna.drabczyk2@pk.edu.pl (A.D.); kinga.korniejenko@pk.edu.pl (K.K.); beata.figiela@pk.edu.pl (B.F.); 2 “Raluca Ripan” Institute for Research in Chemistry, Babes-Bolyai University, 30 Fantanele Street, 400294 Cluj-Napoca, Romania

**Keywords:** geopolymer nanocomposites, sustainable development, inorganic nanoparticles, carbon nanotubes, graphene, nanoclay

## Abstract

The demand for geopolymer materials is constantly growing. This, in turn, translates into an increasing number of studies aimed at developing new approaches to the methodology of geopolymer synthesis. The range of potential applications of geopolymers can be increased by improving the properties of the components. Future directions of studies on geopolymer materials aim at developing geopolymers showing excellent mechanical properties but also demonstrating significant improvement in thermal, magnetic, or sorption characteristics. Additionally, the current efforts focus not only on the materials’ properties but also on obtaining them as a result of environment-friendly approaches performed in line with circular economy assumptions. Scientists look for smart and economical solutions such that a small amount of the modifier will translate into a significant improvement in functional properties. Thus, special attention is paid to the application of nanomaterials. This article presents selected nanoparticles incorporated into geopolymer matrices, including carbon nanotubes, graphene, nanosilica, and titanium dioxide. The review was prepared employing scientific databases, with particular attention given to studies on geopolymer nanocomposites. The purpose of this review article is to discuss geopolymer nanocomposites in the context of a sustainable development approach. Importantly, the main focus is on the influence of these nanomaterials on the physicochemical properties of geopolymer nanocomposites. Such a combination of geopolymer technology and nanotechnology seems to be promising in terms of preparation of nanocomposites with a variety of potential uses.

## 1. Introduction

Geopolymers belong to the group of the fastest-growing polymeric materials. The interest in these inorganic ceramic materials is constantly growing. This is a result of their properties, including acid resistance, porosity, low drying shrinkage, as well as high strength, due to which geopolymers are widely investigated for potential application in the construction industry (e.g., repairing roads, bridges, or other infrastructure) [1,2,3]. Importantly, geopolymers are also used as a substitute for Portland cement. Compared to Portland cement, geopolymers are cheaper, and their fabrication releases less carbon dioxide [4,5].

The use of geopolymers in wastewater treatment (for removing heavy metals) [6,7,8,9], soil stabilization [10], carbon capture and storage [11], or as protective coatings [12,13] is also being investigated. Furthermore, studies on the application potential of these materials for biomedical purposes including tissue engineering [14] or drug delivery systems [15] have also been performed.

**Figure 1:**
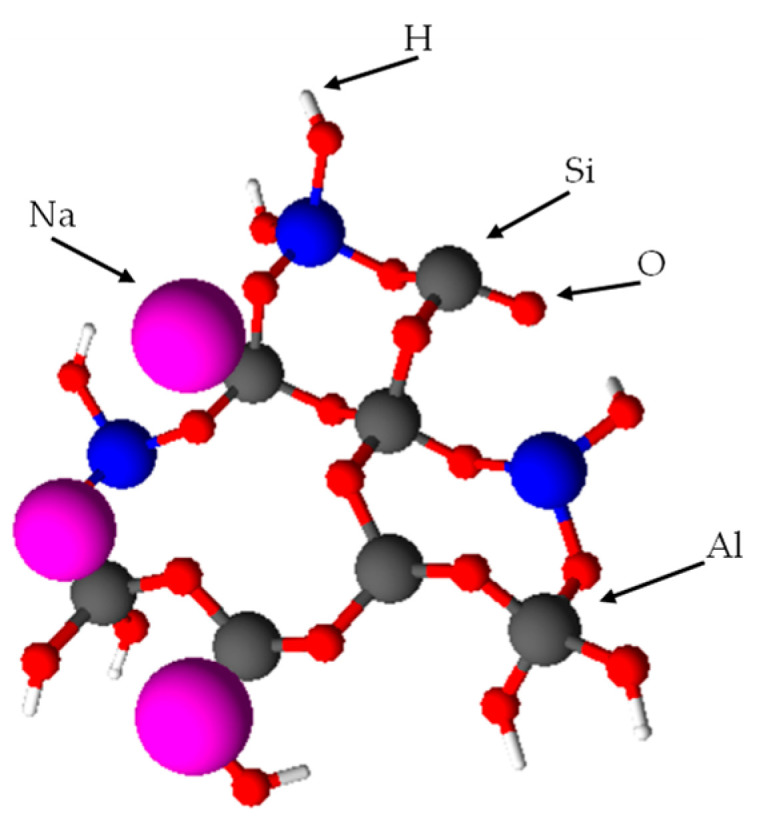
The scheme of the geopolymer framework [16].

The structure of geopolymers consists of networks of inorganic molecules combined by means of covalent bonds. The scheme of the geopolymer framework is presented in Figure 1.

Due to the increasing demand for these materials as well as placing ever higher demands on them, many investigations are being performed to develop geopolymers showing the most beneficial physicochemical properties. Particular attention is paid to studies on geopolymer nanocomposites; the term “*geopolymer nanocomposite*” refers to the hybrid material made up of a combination of a geopolymer matrix and a nanosized modifying agent. It was demonstrated in many literature reports that the incorporation of various nanomaterials into geopolymer matrices results in significant improvement of their functional properties, including, e.g., compressive strength and stiffness [17,18]. Hence, in this review, a number of geopolymer nanocomposites incorporated with selected nanomaterials are presented, wherein the main focus is on the influence of nano-sized materials on their physicochemical properties.

**Figure 2:**
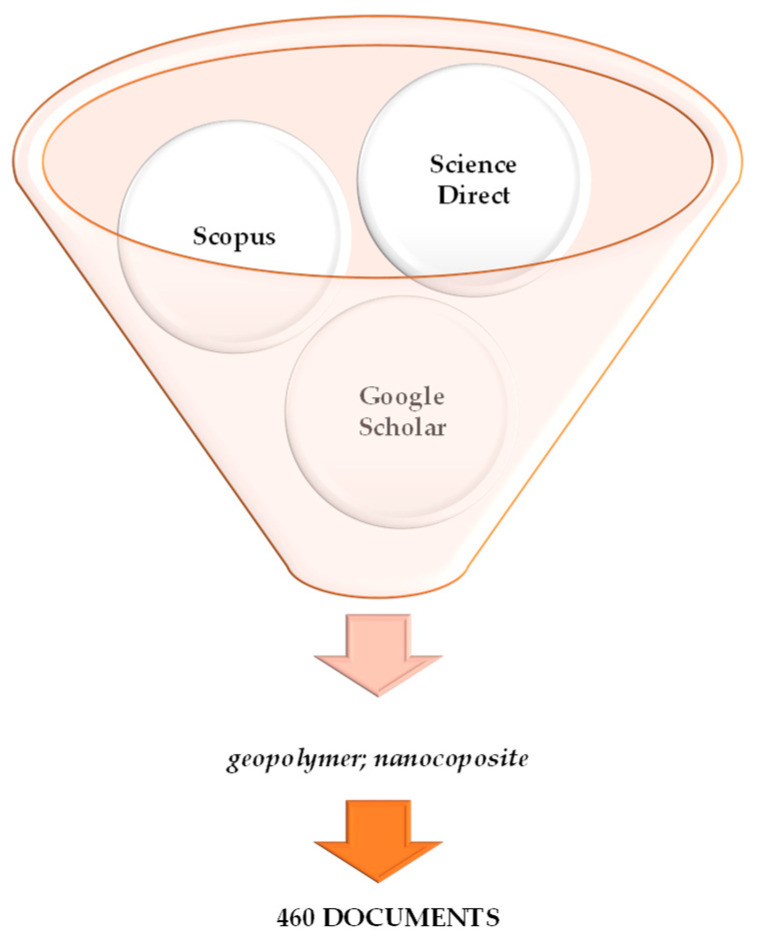
Search results for the terms “*geopolymer*” and “*nanocomposite*” in scientific databases.

The review was prepared employing scientific databases such as ScienceDirect, Scopus, and Google Scholar. The Scopus database was used as a starting point. The following keywords were applied at the beginning: “*geopolymer*” and “*nanocomposite*” combined together. The research results show 460 documents—this is schematically presented in Figure 2.

**Figure 3:**
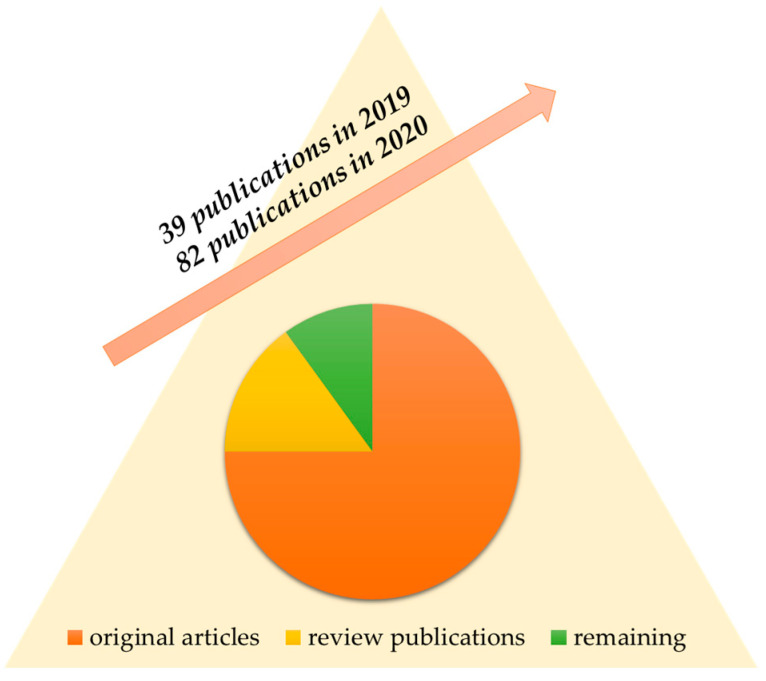
Search results for the terms “*geopolymer*” and “*nanocomposite*” in scientific databases from 2019 and 2020 including the type of the article.

Because of the very fast development in the area of nanocomposites, the current review is focused on the most recent publications, especially within the last 3 years, when rapid increases were noted—for comparison, there were 39 publications in 2019 and 82 in 2020, which is presented in Figure 3.

Nevertheless, some older publications of high importance on the topic were also used. It should be noted that most (>75%) of the published works are original articles, whereas less than 15% are review publications. Amongst the review publications, there is a lack of articles that discuss the problem of geopolymer nanocomposites in the context of a sustainable development approach. This article is the answer to this literature gap. The main objective of this review is to present examples of possible incorporations of geopolymer matrices with nano-sized additives. Developed nanocomposites constitute promising materials obtained using waste products and nanomaterials. Such a combination results in forming a material with enhanced properties compared to unmodified geopolymer matrices.

This review article can help the reader in several ways. Firstly, it presents the current state of knowledge on geopolymers and nanocomposite geopolymers, including their various applications and potential uses. The reader can learn about the latest research in this field and understand the benefits of using nanomaterials in geopolymers. Secondly, this article can help the reader understand the complexity of the processes occurring in geopolymers and nanocomposites, as well as their physicochemical properties. The reader can learn about the factors that influence these properties and the methods used to improve them. Thirdly, this article can help the reader understand the concept of sustainable development in the context of using nanocomposite geopolymers. The reader can learn about the materials used to produce these composites and the benefits of their use, such as waste utilization and reduction of carbon dioxide emissions.

Overall, this review article on nanocomposite geopolymers can help the reader understand the latest trends and research results in this field, as well as identify potential applications of these materials.

## 2. Nanomaterials as Modifiers of Geopolymer Composites

Geopolymerization occurs by mixing a mineral or waste material with an alkaline solution as a function of time and temperature. By adding nanoparticles to the matrix, the alkaline solution is immobilized and the pores between the grains of the raw material are filled, the so-called filler effect [19]. Nanoadditives increase the binding strength of the mixture and participate in pozzolanic reactions, which cause the formation of hydrates of calcium silicates [20].

The following nanoadditives were investigated in geopolymer matrices: nanosilica [21,22], nanocellulose [23,24,25], nanoaluminum [26], nanographite [27], and nano-CaCO_3_ [28]. The performance of geopolymer composites largely depends on the uniform distribution of nanoadditives in the matrix and their reaction with the matrix.

Among these nanoparticles, nanosilica and nanocellulose are the most popular in research due to their unique properties. Nanocellulose seems to be an excellent reinforcing agent in various types of materials, especially in composites. The use of nanocellulose to replace, for example, synthetic fibers, leads to the production of a more environmentally friendly material. Nanocellulose, due to its origin and availability, can be successfully used in materials at low concentrations up to several %, due to the interaction between the matrix, e.g., polymer, and nanoparticles [29].

In Table 1 a summary of the influence of selected nanoadditives on properties of geopolymer nanocomposites has been presented.

The use of nanoadditives in geopolymer matrices has a large impact on the proper-ties of the finished material. The basic type of properties most often determined in geo-polymers are mechanical properties, namely compressive strength and bending strength [36].

Research shows that the addition of nano-SiO_2_ to 2% wt. increases the compressive strength by up to 80% compared to geopolymer based on unmodified fly ash cured at room temperature [37]. Subsequent studies with the addition of nanosilica were carried out on geopolymer samples with a metakaolin matrix. Also, an improving effect of the nanoadditive on compressive strength was demonstrated, both for mortar cured at 80 °C (increase by 40%) and at 20 °C (increase by 56%), compared to the material without nanosilica introduced into the mix [38].

A limitation in the constructional use of geopolymers is their quasi-brittle cracking. Therefore, along with the introduction of nano-SiO_2_, scientists introduce various types of fibers into the geopolymer matrix to improve the structural properties and durability of geopolymers [30]. Thus, the introduction of a combination of PVA fibers and nanosilica particles to the fly ash and the activator increased the compressive strength (by 26% on average) and the fracture toughness compared to the reference sample [39].

The use of the addition of aluminum nanooxide to improve mechanical strength also brings good results. Research by Phoo-ngernkham et al. showed that for 1% wt. of nano-Al_2_O_3_ content, the compressive strength of geopolymer paste with fly ash increases by about 43%. Optimally, to maintain the best strength, it is not to exceed the addition of 3% wt. of nanoparticles [40].

A positive effect on the strength properties was also noted for calcium carbonate nanoparticles, especially for thermal hardening of the geopolymer for 24 h (increase in strength by approx. 60%) [41].

Research on geopolymers can take place at various stages of the process of their creation. One of these is the course of geopolymerization. In its course, nanoparticles can have different effects. The addition of nanosilica to fly-ash-based geopolymers increases the activity of the matrix material, and thus accelerates the geopolymerization process, increasing the length of the C–S hydrogen gel chain, which gives the effect of filling with small particles. Finally, the fly ash and nano-SiO_2_ geopolymerize to form a three-site reticular inorganic gel material with Si–Al–O cross-linking [42].

The higher the nano-SiO_2_ content, the shorter is the setting time. This is mainly due to the unique “surface effect” of nano-SiO_2_. The high surface area of nano-SiO_2_ has high activity and surface energy, which enriches the surrounding free phase on the surface of the nanomaterial, thus accelerating the geopolymerization process. At the same time, the concentration of monomers such as -OSi(OH)_3_-, -OSi(OH)_2_O- in the system accelerates the hydration and condensation hardening of fly ashes [43].

For construction materials, the important properties are resistance to freezing and propagation. It is no different in the case of geopolymer materials, where this property can be determined on the basis of the coefficient of loss of geopolymer compressive strength [44].

Most of the current research [3,18] on geopolymers reinforced with various nanomaterials is at an early stage, and little research has focused on their value for engineering applications and their sustainable aspects. Moreover, dynamic properties and properties after increased temperature are important for the safety of concrete structures. However, the current research [18,45] results focus on the static mechanical properties of nano-SiO_2_- and nanocellulose-modified geopolymer composites, and little research concerns dynamic mechanical properties and properties after elevated temperature. Therefore, further research on the dynamic properties of nanocomposites before and after their treatment with elevated temperature is needed to accelerate the development of geopolymers enriched with nanoadditives and increase their utility value. In Table 2. the impact of the introduction of the nanoclay nanoparticles into the geopolymers based on volcanic tuff and fly-ash slag has been reported.

**Figure 4:**
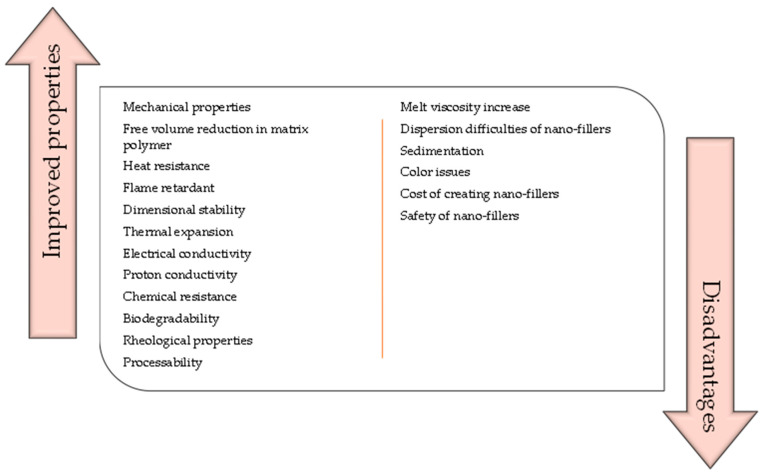
Selected advantages and disadvantages of nanocomposites.

Nanocomposite materials show both advantages and disadvantages [6,7,8] which are presented in Figure 4.

Despite of some disadvantages of nanocomposites, they show many interesting properties which result in growing interest in studies on these materials. Some examples of such nanocomposites are presented in the following section.

## 3. Geopolymer Nanocomposites Reinforced with Selected Nanomaterials

### 3.1. Geopolymer Nanocomposites Reinforced with Carbon Nanotubes

Geopolymer nanocomposites reinforced with carbon nanotubes (CNTs) have gained significant attention in recent years due to their unique mechanical, thermal, and electrical properties. Carbon nanotubes are cylindrical carbon molecules that have exceptional mechanical, electrical, and thermal properties. In this subsection, the synthesis, properties, and applications of CNT-containing geopolymer nanocomposites are described. There are two main approaches used to these nanocomposites: in situ and ex situ methods. In situ methods involve the addition of carbon nanotubes during the synthesis of the geopolymer matrix. In turn, ex situ processes involve the addition of pre-synthesized carbon nanotubes to the geopolymer matrix. In situ methods are advantageous because they result in a more homogeneous distribution of CNTs within the geopolymer matrix. However, they require careful control of the synthesis parameters, such as pH and temperature, to prevent the degradation of the carbon nanotubes. Ex situ methods are easier to control, but the dispersion of the carbon nanotubes within the geopolymer matrix is not as uniform [48,49,50].

Geopolymer nanocomposites with CNTs show excellent mechanical properties, including high tensile strength, compressive strength, and flexural strength. The addition of carbon nanotubes improves the fracture toughness and reduces the brittleness of the geopolymer matrix. The high aspect ratio of carbon nanotubes also enhances the load transfer between the matrix and the reinforcement [51,52,53]. Additionally, CNT-containing geopolymer nanocomposites have superior thermal properties compared to traditional composites. The addition of carbon nanotubes enhances the thermal conductivity of the geopolymer matrix, which is important for applications such as thermal management. The thermal stability of the geopolymer matrix is also improved by the addition of carbon nanotubes. The described nanocomposites also demonstrate excellent electrical properties, including high electrical conductivity and low dielectric constant. These properties make them suitable for applications such as electromagnetic shielding and energy storage [54,55,56].

Geopolymer nanocomposites containing CNTs have potential applications in various industries, including aerospace, automotive, and construction. In the aerospace industry, these materials can be used to manufacture lightweight, high-strength components. In the automotive industry, they can be used to manufacture components with improved fuel efficiency and reduced emissions. In the construction industry, they can be used to manufacture high-strength, durable building materials. Overall, this type of nanocomposite is a promising class of materials with superior mechanical, thermal, and electrical properties. The addition of carbon nanotubes enhances the properties of geopolymer matrices, making them suitable for a wide range of applications. However, further research is needed to optimize the synthesis parameters and to investigate the long-term stability of these materials [57,58,59,60].

### 3.2. Geopolymer Nanocomposites Containing Graphene and Graphene Oxide

Geopolymer nanocomposites containing graphene and graphene oxide have been extensively studied in recent years due to their unique properties and potential applications in various industries. Graphene and graphene oxide are two-dimensional carbon-based materials that have excellent mechanical, electrical, and thermal properties. Several methods are used to synthesize geopolymer nanocomposites containing graphene and graphene oxide, including in situ and ex situ methods, which involve the addition of graphene or graphene oxide during the synthesis of the geopolymer matrix (in situ) and the addition of pre-synthesized graphene or graphene oxide to the geopolymer matrix (ex situ). More uniform distribution of graphene or graphene oxide within the composite matrix is achieved via in situ processes which, in turn, require strict control of the process conditions (temperature, pH etc.) [61,62,63,64,65].

Geopolymer nanocomposites with graphene and graphene oxide exhibit excellent mechanical properties, including high tensile strength, compressive strength, and flexural strength. The addition of graphene or graphene oxide improves the fracture toughness and reduces the brittleness of the geopolymer matrix. The high aspect ratio of graphene or graphene oxide also enhances the load transfer between the matrix and the reinforcement. Importantly, such nanocomposites demonstrate superior thermal properties, enhanced thermal conductivity, and thermal stability compared to composites without these nanoadditives. Moreover, graphene and graphene oxide nanocomposites exhibit excellent electrical properties, including high electrical conductivity and low dielectric constant, which make them useful in terms of their application in electromagnetic shielding and energy storage [66,67,68,69,70].

**Figure 5:**
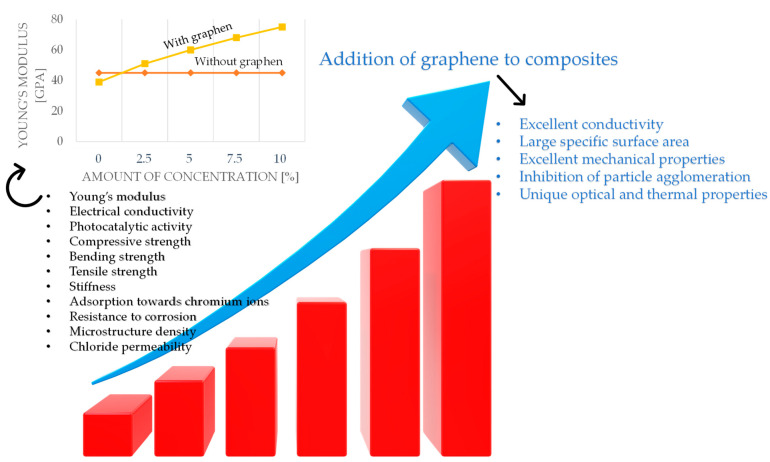
The impact of graphene on selected characteristics of geopolymers.

The overall influence of graphene on selected physicochemical characteristics of geopolymers is illustrated in Figure 5.

The discussed nanocomposites have potential applications in various industries, including aerospace, automotive, and construction. In the aerospace industry, these materials can be used to manufacture lightweight, high-strength components. In the automotive industry, they can be used to manufacture components with improved fuel efficiency and reduced emissions. In the construction industry, they can be used to manufacture high-strength, durable building materials. Such wide range of applications of the nanocomposites is due to their superior electrical, mechanical, and thermal properties [71,72,73,74].

### 3.3. Geopolymer Nanocomposites Reinforced with Nanoclay

Geopolymer nanocomposites reinforced with nanoclay are a type of material that has gained significant attention in recent years. These composites combine the benefits of geopolymer technology, such as high strength and durability, with the reinforcing effects of nanoclay particles, resulting in improved mechanical, thermal, and chemical properties. The synthesis of geopolymer nanocomposites reinforced with nanoclay can be achieved using various methods, although the most common is the in situ method, where nanoclay particles are added during the synthesis of the geopolymer matrix [75,76].

Geopolymer nanocomposites containing nanoclay demonstrate improved mechanical properties such as enhanced strength, stiffness, and toughness. This is due to the high aspect ratio of the nanoclay particles, which reinforce the geopolymer matrix by acting as a physical barrier against crack propagation. The addition of nanoclay particles also improves the thermal stability of the geopolymer matrix, making it suitable for high-temperature applications. Additionally, the presence of nanoclay particles can improve the chemical resistance of geopolymer nanocomposites, providing protection against chemical degradation [77,78].

Importantly, nanoclay-containing geopolymer nanocomposites have applications in the construction, automotive, and aerospace, industries. In the construction industry, these materials can be used to manufacture high-strength, durable building materials, such as pipes and panels. In turn, in the aerospace industry, they can be used to manufacture lightweight components for aircraft and space vehicles. In the automotive industry, they can be used to manufacture components with improved fuel efficiency and reduced emissions [79,80,81].

Despite the numerous benefits of geopolymer nanocomposites reinforced with nanoclay, there are still some challenges that need to be addressed. One of the primary challenges is the cost of producing these materials, as nanoclay particles are relatively expensive. Furthermore, the optimization of the synthesis parameters, such as pH and temperature, is crucial to ensure consistent and reproducible results [82,83].

In the future, research will focus on the development of more cost-effective methods for producing the described geopolymer nanocomposites. Additionally, further research is needed to investigate the long-term stability of these materials under various environmental conditions, such as exposure to UV radiation and humidity. The development of new applications for geopolymer nanocomposites reinforced with nanoclay will also be an area of focus in the coming years. Geopolymer nanocomposites with nanoclay are a promising class of materials with improved mechanical, thermal, and chemical properties. These materials have the potential for various applications in the construction, aerospace, and automotive industries. Although there are still some challenges that need to be addressed, the development of cost-effective methods for producing these materials and the investigation of their long-term stability will drive future research in this field [84,85,86].

### 3.4. Geopolymer Nanocomposites with Magnetic Nanoparticles

The incorporation of magnetic nanoparticles into geopolymer nanocomposites has added a new dimension to the potential applications of these materials, making them suitable for a range of innovative technologies, including drug delivery, environmental remediation, and magnetic separation. The synthesis of geopolymer nanocomposites with magnetic nanoparticles can be achieved using both in situ and ex situ approaches [61].

Geopolymer nanocomposites with magnetic nanoparticles exhibit improved magnetic properties such as enhanced magnetic susceptibility, magnetic field response, and magnetic saturation. This is due to the presence of magnetic nanoparticles, which are capable of responding to external magnetic fields. Additionally, the incorporation of magnetic nanoparticles does not significantly affect the mechanical, thermal, and chemical properties of geopolymer nanocomposites [87,88,89].

Magnetic-nanoparticle-containing geopolymer nanocomposites have the potential for various applications in the fields of drug delivery, environmental remediation, and magnetic separation. In the field of drug delivery, these materials can be used as drug carriers, which can be magnetically guided to specific sites in the body. In the field of environmental remediation, they can be used to remove contaminants from water and soil by magnetically separating them from the environment. In the field of magnetic separation, they can be used to separate and purify magnetic materials from non-magnetic materials [90,91].

In spite of the many benefits of the described nanocomposites, there are still some challenges that need to be addressed. One of the primary challenges is the optimization of the synthesis parameters, such as pH and temperature, to ensure consistent and reproducible results. Additionally, the cost of producing these materials needs to be reduced to make them more economically feasible [92,93,94].

In the future, research will focus on the development of more cost-effective methods for producing geopolymer nanocomposites with magnetic nanoparticles. Additionally, further research is needed to investigate the long-term stability of these materials under various environmental conditions, such as exposure to humidity and corrosive environments. The development of new applications for geopolymer nanocomposites with magnetic nanoparticles will also be an area of focus in the coming years. Geopolymer nanocomposites with magnetic nanoparticles constitute an interesting class of materials showing enhanced magnetic properties, which makes them appropriate for a range of innovative technologies. However, the development of cost-effective methods for producing these materials and the investigation of their long-term stability will drive future research in this field. The potential applications of these materials in drug delivery, environmental remediation, and magnetic separation make them an exciting area of research in the field of materials science [95,96,97].

### 3.5. Geopolymer Nanocomposites Reinforced with Titanium Dioxide Nanoparticles

Geopolymer nanocomposites reinforced with nanoparticles, such as titanium dioxide (TiO_2_), have been developed to enhance their mechanical, thermal, and chemical properties. The synthesis of geopolymer nanocomposites reinforced with TiO_2_ can be achieved using the same approaches as in the case of the nanocomposites described in previous subsections of this paper, i.e., ex situ and in situ [98].

Geopolymer nanocomposites containing TiO_2_ nanoparticles exhibit improved mechanical, thermal, and chemical properties such as enhanced flexural strength, compressive strength, thermal stability, and resistance to chemical attack. This is due to the presence of TiO_2_ nanoparticles, which act as reinforcement agents, filling the gaps between the geopolymer matrix and improving its mechanical properties. Additionally, the incorporation of TiO_2_ nanoparticles can improve the photocatalytic activity of geopolymer nanocomposites. These nanocomposites have the potential for various applications in the fields of construction, environmental remediation, and energy storage. In the field of construction, these materials can be used to produce high-performance, durable building materials, such as concrete and mortar. In the case of environmental remediation, they can be used to remove contaminants from water and soil by photocatalytic degradation, while in the area of energy storage, they may be applied to produce high-performance supercapacitors [99,100,101].

Considering further studies on these nanocomposites, aspects such as pH and temperature employed during their synthesis should be investigated so as to obtain desirable results. Furthermore, their long-term stability and durability under various conditions also need to be verified. In the future, research will focus on the development of more cost-effective methods for producing geopolymer nanocomposites reinforced with TiO_2_. Additionally, further research is needed to investigate the effects of TiO_2_ particle size, shape, and surface modification on the properties of geopolymer nanocomposites. So far, the incorporation of TiO_2_ in geopolymer matrix has shown to improve the mechanical properties such as compressive strength, flexural strength, and fracture toughness. This improvement is due to the enhanced interfacial bonding between the geopolymer matrix and TiO_2_ particles. Additionally, TiO_2_ improves the thermal stability of geopolymer nanocomposites [102,103,104].

In conclusion, geopolymer nanocomposites reinforced with TiO_2_ have shown significant potential for various applications in the fields of construction, biomedicine, and environmental remediation. The development of these materials will undoubtedly contribute to the advancement of materials science, particularly in the search for sustainable and eco-friendly materials [105,106].

### 3.6. Geopolymer Nanocomposites Reinforced with Nanosilica

The addition of nanoparticles, such as nanosilica, to geopolymer matrices has been studied to improve their properties further. Nanosilica can be synthesized using various methods, including sol-gel, precipitation, and hydrothermal methods. The most commonly used method for the synthesis of nanosilica is the sol-gel method, which involves the hydrolysis and condensation of silicon alkoxides. The resulting nanosilica particles can then be incorporated into geopolymer matrices using in situ or ex situ methods [61].

Incorporation of nanosilica into geopolymer matrices has been shown to improve their mechanical, thermal, and chemical properties. The incorporation of nanosilica increases the density and reduces the porosity of the geopolymer matrix, resulting in improved mechanical properties such as compressive strength, flexural strength, and fracture toughness. Nanosilica also enhances the thermal stability of geopolymers and improves their chemical resistance, making them more resistant to acid and alkali attack. As a result, the addition of nanosilica to geopolymer matrices has expanded their potential applications in various fields, such as construction, energy, and environmental remediation. In the field of construction, geopolymer-based composites reinforced with nanosilica have been shown to produce high-performance materials, such as mortar and concrete. In the energy sector, geopolymer-based materials reinforced with nanosilica can be used for energy storage, such as supercapacitors. In environmental remediation, geopolymer-based composites reinforced with nanosilica have been used to remove heavy metals and organic pollutants from contaminated water [107,108,109,110].

Importantly, introduction of nanosilica into geopolymer matrices has been shown to improve their chemical, mechanical, and thermal properties. The incorporation of nanosilica increases the density and reduces the porosity of the geopolymer matrix, resulting in improved mechanical properties such as compressive strength, flexural strength, and fracture toughness. Additionally, the improved thermal stability and chemical resistance make nanosilica-modified geopolymer matrices suitable for a wide range of applications in construction, energy storage, and environmental remediation [111,112,113,114].

Despite the potential benefits, there are still challenges that need to be addressed. With continued research, it is likely that nanosilica-modified geopolymer matrices will become even more versatile and cost-effective, making them a promising alternative to traditional cement-based materials. Overall, the addition of nanosilica to geopolymer matrices shows great promise for enhancing the properties and expanding the potential applications of these inorganic materials [115,116].

The influence of nanomaterials on selected properties of geopolymer nanocomposites is summarized in Table 3.

Geopolymers are ceramic materials obtained from mineral raw materials such as fly ash or slag. Nanomaterial additives are used to improve the mechanical and thermal properties of geopolymers.

The mechanism of action of nanomaterial additives on the structure and properties of geopolymers is complex and depends on the type of nanomaterial, its concentration, the method of introduction into the geopolymer structure, and the conditions during production and curing.

In research conducted by De Silva et al. [175], the addition of carbon nanotubes to a geopolymer mixture increased its compressive and flexural strength by approximately 29% and 21%, respectively. Similar results were obtained by using silica nanoparticles [176] and aluminum oxide [177].

Nanomaterial additives can affect the structure of geopolymers by increasing the number of crosslinking bonds, reducing porosity, and improving homogeneity. In addition, nanomaterials can affect the phase structure of geopolymers and their thermal decomposition [178].

However, it is worth noting that the results of studies on the impact of nanomaterials on the properties of geopolymers are varied and not always clear. The introduction of nanomaterials into geopolymer mixtures requires further research and optimization of production processes to obtain materials with desired properties.

## 4. Sustainability of Geopolymer Nanocomposites

Geopolymer nanocomposites are a type of sustainable composite material that has been gaining increasing attention in recent years. These materials are composed of a geopolymer, a cementitious material that is synthesized from industrial waste or natural minerals, and nanoparticles, which are added to improve the mechanical, thermal, and electrical properties of the resulting material [179].

There has been significant research into the sustainability of geopolymer nanocomposites, with many studies focusing on their environmental impact and long-term durability. One key advantage of geopolymer nanocomposites is their low carbon footprint, as they can be produced using industrial waste materials such as fly ash, blast furnace slag, and metakaolin. This reduces the amount of waste sent to landfills and decreases the need for traditional Portland cement production, which is responsible for a significant amount of global carbon emissions [179,180].

In addition to their sustainability, geopolymer nanocomposites also offer several technical advantages over traditional cement-based materials. They have been found to have excellent mechanical properties, including high compressive and flexural strength, as well as good resistance to fire and chemicals. Furthermore, they exhibit good thermal stability and can be used in high-temperature applications. Recent studies have also investigated the use of geopolymer nanocomposites in various engineering applications, including the construction of bridges, roads, and buildings. These studies have demonstrated the potential of these materials to provide sustainable and durable solutions in the built environment. Overall, the literature suggests that geopolymer nanocomposites offer a promising avenue for the development of sustainable composite materials. Ongoing research is focused on optimizing their properties and exploring new applications, with the aim of further reducing the environmental impact of the construction industry [181].

**Figure 6:**
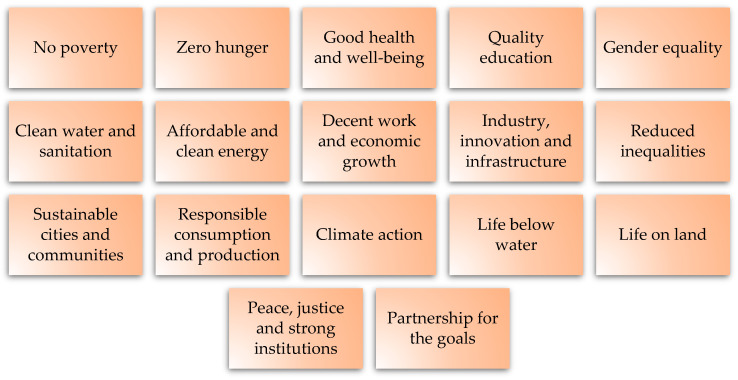
Sustainable development goals.

Analyzing the sustainable development goals, we may find that the geopolymer nanocomposites are coherent with all three main areas of this approach: environment, society, and economy [182,183]. The sustainable development approach is based on 17 goals that are presented in Figure 6.

Geopolymers belong to a group of rapidly developing materials. Research on geopolymers is performed while simultaneously paying attention to the lack of negative impact on the environment and considering sustainable development goals. These materials have the potential to reduce negative environmental impact due to their many positive features. Firstly, nanogeopolymer composites are made from natural materials such as volcanic ash, which is a waste material from power plants or cement factories, meaning they are less harmful to the environment than traditional building materials such as cement. Secondly, nanogeopolymer composites have a smaller environmental impact during production. Unlike cement production, which is associated with high carbon dioxide emissions, the production of nanogeopolymer composites is much more energy efficient and has a lower environmental impact. Thirdly, nanogeopolymer composites are more durable and resistant to weather conditions than traditional building materials. As a result, their lifespan increases, meaning there is less need for replacement, which translates to a smaller amount of construction waste. Finally, nanogeopolymer composites can be used to produce materials that are much lighter and smaller in size than building materials, which can contribute to a reduction in material and energy consumption during the construction process. In summary, nanogeopolymer composites are environmentally friendly building materials that have the potential to reduce negative environmental impacts compared to traditional building materials. Newly designed functional materials may in turn contribute to the growth of the industrial sector in which they are applied and thus to the overall economic growth. From an environmental point of view, the most significant feature of geopolymers is the low emission of greenhouse gases during production, including low demand on energy. The main benefits from this fact can be achieved through the large-scale application in the building industry as infrastructure projects. It is also worth mentioning that the fabrication of geopolymer-based concretes and cements produces significantly lower carbon dioxide emissions compared to the fabrication of Portland cement, which is undoubtedly favorable in terms of environmental protection and phenomena affecting climate change (including the greenhouse effect) [184,185,186,187,188,189].

However, given that there are many limitations in such applications, enhancing the geopolymer characteristics by nanoadditives could be a promising solution. Today, the building industry looks for smart solutions, such as self-condition monitoring, self-curing properties, energy storage, energy harvesting, etc. Some of them can be obtained by nano-components. For example, graphene–geopolymer composites show a piezoresistive effect, which can be used for self-condition monitoring of building materials. Furthermore, the addition of graphene pellets can also influence the energy-storage or energy-harvesting properties of geopolymers. The nanoparticles also allow for ‘self-cleaning’ properties. The addition of nano titanium oxide in construction materials, thanks to the photocatalyst process, helps to decompose a wide range of air pollutants and to remove harmful bacteria. In addition, this additive also produces one more desirable sustainable effect—improving the air quality in urban areas [179,180,181].

It is worth noting that the mentioned properties of geopolymer nanocomposites are also connected with the area of economy [190,191] and goals connected with sustainable cities as well as industry and infrastructure [182,183]. Research shows that geopolymer nanocomposite not only have better mechanical properties, but first of all better durability [189,192]. For example, nano titanium dioxide decreases carbonation depth [192] and raises chloride and sulfate resistance through the formation of hydrates and the filling effect [193]. In turn, nanosilica significantly attenuates water absorption [194] and significantly enhances the material resistance to acid environments and limiting surface deterioration thanks to the decreased porosity and increased density of the geopolymer [195]. For infrastructure applications, particularly interesting are different forms of nanocarbon [196]. For example, carbon nanotubes improved structural and mechanical properties and increased resistance in sulfate attack [197,198]. Moreover, the proper design of the composites gives the possibility of self-condition monitoring, which can significantly decrease the cost of maintaining infrastructural composition that can be provided only on-demand (signal received from material) [199].

Firstly, due to the fact that nanocomposites are more durable and resistant to weather conditions than traditional building materials, they can significantly reduce costs associated with maintenance and repairs. As a result, property owners can save a significant amount of money in the long run. Secondly, the production of nanocomposites can be much more energy-efficient than the production of traditional building materials such as cement. This means that production costs can be lower, which can lead to lower costs for end consumers. Thirdly, nanocomposites can be used to produce materials that are lighter in weight and smaller in size than traditional building materials, which can help reduce transportation and handling costs during the construction process. In summary, nanocomposites have the potential to reduce costs in the construction industry by reducing maintenance and repair costs, lowering production and transportation costs, and reducing material and energy consumption during the construction process [200,201,202,203,204,205,206].

An important aspect of the economy is also the sustainable development goal associated with energy. Geopolymers themselves are well known as materials that allow for low energy consumption [174,207]. The usage of nanocomponents additionally strengthens these properties. Firstly, due to increasing the mechanical properties, a significantly lower amount can be used. Increasing the mechanical properties of a material has several benefits, one of which is that it allows for the use of a lower amount of the material while still achieving the desired level of performance. This can result in cost savings and can also be beneficial for the environment by reducing the amount of resources required to produce the material. Additionally, using a lower amount of material can also lead to a reduction in the amount of waste generated, further contributing to environmental sustainability. Therefore, improving the mechanical properties of materials can have wide-ranging benefits, both economic and environmental. In particular, a very good effect is obtained by joining different nanoparticles and fibers in geopolymer composites [194,208,209] as well as by combining nanoparticles with each other or with microparticles [210]. Furthermore, the addition of nanocomponents, such as silica carbon whiskers, improves the mechanical properties and toughness of geopolymers [174,211]. It results in the possibility of using a lower amount of material to obtain the same strength parameters for particular construction. The element that should also be considered in a case of materials is a possibility of its production from waste or renewable raw materials. This is a strong point of geopolymer nanocomposites. Among the most popular materials for the geopolymerization process are waste products from the energy industry such as fly ash and slags [212]. Moreover, among the possibilities considered in the last a few years are natural microfibers, such as cellulose nanofibrils or nanocrystalline cellulose [213]. This type of additive is more environmentally friendly than synthetic nanofibers, but should bring similar benefits.

The research and development of geopolymer materials is driven by the sustainability factor. Although replacing concrete on a large scale is far from implementation, geopolymer composites are increasingly being used in niche areas. Geopolymer nanocomposites are particularly interesting because of their advanced properties that are useful in many areas. They are coherent with all three main areas of sustainable development goals: environment, society, and economy. The fabrication of geopolymer materials is in line with responsible production, innovation, and waste management, and reduces the carbon footprint. Geopolymers emit low levels of greenhouse gases during production, making them favorable for environmental protection. The addition of nanoadditives enhances the characteristics of geopolymers and offers promising solutions to overcome the limitations of applications. Graphene–geopolymer composites show piezoresistive effects that can be used for self-condition monitoring of building materials, while nano titanium oxide helps to decompose air pollutants and improve air quality in urban areas. Geopolymer nanocomposites have better mechanical properties and durability, making them suitable for infrastructure and shielding applications. They can resist biocorrosion and algae and fungi formation, and they have self-healing properties. Therefore, geopolymer nanocomposites have a significant impact on sustainable cities, industry, and infrastructure [213,214,215,216,217,218,219,220,221,222,223].

## 5. Conclusions and Perspectives

The research direction in the field of polymers focuses on developing various approaches for their synthesis and modification in line with sustainable development goals. Geopolymer fabrication can use waste products, which is considered economically and environmentally friendly and in line with waste management and responsible production approaches. Studies are being performed to modify geopolymer matrices to enhance their mechanical properties or provide new properties such as magnetism or sorption. Nanomaterials are being increasingly used as potential modifying agents of geopolymers. Even a small amount (lower than 1.0% wt.) of nanomaterials added to the geopolymer matrix can significantly improve its functional properties. Nanomaterials can enhance mechanical properties such as compressive strength, flexural strength, and toughness, as well as affect characteristics such as permeability, water adsorption, porosity, or thermal properties. The combination of a geopolymer matrix with waste products and nano-sized materials can lead to the development of innovative functional materials and affect economic growth, especially in the construction industry.

Geopolymers have potential for sustainable development due to their low emission of greenhouse gases and low energy demand during production. The main conclusion drawn from these findings is that the use of geopolymer materials combined with waste products and nanomaterials has the potential to lead to the development of innovative and sustainable functional materials. This approach aligns with sustainable development goals by minimizing negative environmental impacts and promoting responsible waste management and production. The incorporation of nanomaterials into geopolymer matrices can significantly improve their mechanical and functional properties, making them a promising solution for the construction industry and other sectors. Additionally, geopolymer production is energy-efficient and emits low levels of greenhouse gases, further contributing to sustainable development.

## Figures and Tables

**Table 1:** Overview of nanoadditives used in geopolymers and their impact on properties.

Nanoadditive	Investigated Effect	Source
Nano-SiO_2_	Adding nano-SiO_2_ to fly-ash-based geopolymers can enhance the activity of fly ash, thus accelerating the geopolymerization process, increasing the length of the C–S hydrogen gel chain, and producing a small particle-filling effect. Finally, the fly ash and nano-SiO_2_ geopolymerize to form a three-site reticular inorganic gel material with Si–Al–O cross-linking.	[21]
	The higher the incorporation of nano-SiO_2_, the shorter is the setting time.	[22]
	The dry shrinkage performance of the geopolymer improved, and the improvement due to nano-SiO_2_ was greater than that due to nano-γ-Al_2_O_3_.	[30]
	With an increase in the content of nano-SiO_2_, the freezing–thawing resistance of the geopolymer is gradually strengthened at first and then gradually declines. After mixing with nano-SiO_2_, the geopolymer becomes denser, the pore size inside the material decreases, and the number of pores decreases, which reduces the damage to the internal structure during the freezing–thawing cycle.	[31]
Nanocellulose	Addition of less than 0.5% by weight of nanocellulose crystals promotes mechanical properties; on the other hand, higher concentrations of this additive protect the geopolymer against cracking in unstable curing conditions	[32]
Nanographite	A 3D-printed geopolymer with 1% wt. of NGPs increased the flexural strength by 89% and 46% compared to the same 3D-printed and casted geopolymer without any NGPs, respectively. The same increase for compressive strength was 28% and 12%. Moreover, the geopolymer mix containing 1% wt. of NGPs demonstrated the best shape retention and buildability.	[27]
Nano-CaCO_3_	The use of 3% wt. nano-CaCO_3_ in basalt-fiber-reinforced geopolymer paste presented the highest values of compressive strength and hardness while the use of 2% wt. nano-CaCO_3_ showed the highest values of flexural strength, impact strength, and fracture toughness of composite paste.	[28]
Nano-Al_2_O_3_	Addition of up to 2% wt. nano-Al_2_O_3_ increases the mechanical properties of geopolymer concrete, while the addition of 1%, 2% by weight is optimal for obtaining improvement.	[33,34]
	Addition of 0.75 wt. nano-aluminium improved compressive strength both in the early stage of the test (7 days) and after 28 days. Adding more nanoparticles worsened this property.	[35]

**Table 2:** Properties of geopolymers with nanoadditives at elevated temperatures.

Nanoparticles	Matrix	Investigated Effect	Source
Nanoclay	Volcanic tuff	The compressive strength of the geopolymer with the addition of nanoclay increases with heating up to 300 °C. Above this temperature, it decreases by about 20%.	[46]
	Fly-ash slag	The addition of nanoclay in the amount of 6% wt. improves the compressive strength by 26% in relation to the base sample. In addition, this strength increases proportionally after exposure to a higher temperature of 200 °C; as the temperature increases further, the strength decreases by about 15%.	[47]

**Table 3:** The reported influence of selected nanomaterials on the properties of geopolymer nanocomposites.

Nanoadditive	Observed Effect	Source
Carbon nanotubes	Enhanced compressive strength, flexural strength, and mechanical fracture parameters	[117,118,119,120,121,122,123,124]
	Enhanced Young’s modulus and flexural toughness	[123]
	Decreased sorption properties and setting time	[125]
	Decreased bulk density and porosity	[126]
	Enhanced relative permittivity	[127]
	Enhanced fracture energy, piezoresistive response, and electrical conductivity	[123]
	Increased stiffness and fracture toughness	[128]
	Increased thermal conductivity	[129]
Graphene	Improved electroconductivity, cycling durability, structure stability, and photocatalytic activity	[130]
	Added photocatalytic activity	[131]
	Decreased workability and enhanced compressive strength	[132]
	Enhanced stiffness, toughness, flexural strength, and compressive strength	[133]
Graphene oxide	Enhanced adsorption properties and photocatalytic activity	[134]
	Improved thermal conductivity and permeability	[135]
	Increased compressive strength	[135,136,137,138,139]
	Improved tensile strength and corrosion resistance	[140]
	Enhanced modulus of elasticity, chloride permeability, and microstructure density	[136]
	Enhanced ion-immobilization ability	[139]
	Enhanced flexural strength and fracture toughness	[141,142,143]
Nanoclay	Enhanced compressive and flexural strength	[144,145,146]
	Enhanced rheological properties	[147]
Magnetic nanoparticles	Added magnetic properties	[148,149,150,151]
	Enhanced adsorption properties	[148,150,152]
	Enhanced removal efficiency and high recyclability	[149]
Titanium dioxide	Reduced roughness and swelling ability	[153]
	Reduced porosity	[153,154]
	Enhanced photocatalytic activity	[153,155]
	Enhanced compressive and flexural strength	[153,154,155,156,157]
	More compact structure, enhanced ductility, and greater load-carrying capacity	[157]
	Enhanced adsorption properties	[158]
	Enhanced carbonation resistance; reduced drying shrinkage	[157]
Nanosilica	Enhanced tensile toughness, compressive strength, elastic modulus, and ductility	[159,160,161,162,163,164,165,166]
	Enhanced acid resistance, lower sorption properties	[167]
	Enhanced durability; reduced porosity	[168]
	Decreased flowability and setting time	[161]
	Reduced gas permeability	[164]
	Enhanced thermal stability	[165]
Nano-silicon carbide	Enhanced thermoelectric properties	[169]
Calcium carbonate nanoparticles	Increased hardness, compressive strength, and flexural strength	[170,171]
	Lower water penetration; decreased water adsorption	[171]
	Decreased porosity	[171]
Nanoalumina	Reduced porosity, setting time, optical transmission, and water absorption; enhanced compressive strength	[172]
Nanozirconia	Enhanced compressive strength, ultrasonic pulse velocity, and thermal properties	[173,174]

## Data Availability

The data that support the findings of this study are contained within the article.
